# Editorial: The use of volatile compounds analysis for the assessment of food and beverage quality

**DOI:** 10.3389/fnut.2023.1250634

**Published:** 2023-07-24

**Authors:** Ben de Lacy Costello, Martyna N. Wieczorek, Natalia Drabinska

**Affiliations:** ^1^Centre for Research in Biosciences, School of Applied Sciences, University of the West of England, Bristol, United Kingdom; ^2^Food Volatilomics and Sensomics Group, Department of Food Technology of Plant Origin, Faculty of Food Science and Nutrition, Poznan University of Life Sciences, Poznan, Poland

**Keywords:** flavor, aroma, volatiles, beverages, food, food quality

The study of volatile compounds from foodstuffs is important in assessing food quality, traceability, safety, product authentication, bioactivity and aspects of flavor development (1, 2). To date there have been over 10,000 volatile compounds identified in foods and beverages (3)—more than the number of volatiles currently recorded from humans and their associated microbiome (4). This highlights the intensive research activity in the area of volatilomics and sensomics applied to analyze food quality.

However, despite this large number of volatile compounds only a limited subset of these around 230 key food odorants were found to actively contribute to the odor profile of >200 foods such as beverages, meat products, cheeses, and baked goods (5). This opens up the possibility of using artificial intelligence in the future assessment of food and beverage quality (6), replacing more traditional sensomics techniques which rely wholly or partly on human olfaction. There are many studies which include artificial olfaction and various machine learning and pattern recognition techniques to assess food and beverage quality (6). The Research Topic includes a hybrid study that utilizes an electronic nose in combination with headspace—SPME GC-MS to grade the quality of Moutai-Flavor based Liquor (Wu et al.).

One of the areas that has impacted on this field of research is the development of new and improved analytical techniques such as high resolution mass spectrometry, tandem mass spectrometry, ambient mass spectrometry (7), Direct mass spectrometry techniques such as selected ion flow tube—mass spectrometry (SIFT-MS) (8), Proton Transfer reaction -Mass spectrometry (PTR-MS), SESI-MS (9) and multidimensional chromatographic techniques (10). A paper in the Research Topic uses tandem mass spectrometry to characterize volatile organic compounds in *Allium* species (Qin et al.).

Another area that has developed over the last few decades is the ability to directly sample headspace via techniques such as SPME and thermal desorption. All four of the papers within the Research Topic utilize headspace-SPME as part of their analysis (Qin et al.; Wu et al.; Li et al.; Tian et al.). The SPME technique is very fast and facile to deploy compared to more conventional techniques such as direct headspace analysis or solvent extraction. SPME can be carried out in the field and there are a range of different coatings available commercially which enable tailoring of the extraction to the compounds of interest.

There is a general trend in food science research to look at food as an affordable method for disease prevention (11). Therefore, the main challenge in this research is to understand the interaction of food compounds with genes and the subsequent effects on proteins and metabolites including volatile metabolites.

A similar aim would be to better understand the effect of food on flavor development via these same interactions. A paper in the Research Topic looks to characterize meat quality from two breeds of sheep by analyzing fatty acids and volatile compounds to better understand the mechanism of flavor development (Li et al.).

The direct analysis of flavor compounds which are known to or suspected to impart health benefits in humans is also an important area of ongoing research. There is paper in the Research Topic which looks at the anti-sclerosis effects of the volatile compounds identified in *Allium* species (Qin et al.).

Finally research into flavor alteration using fermentation is a very important topic in both food and beverage science. In this case volatile compounds can be used to assess both the development of flavor and the ongoing process of fermentation. In this Research Topic there is an interesting paper that looks at the influence of *Lactobacillus helveticus* strains on the aromatic flavor of fermented sausages (Tian et al.).

In conclusion the study of volatile compounds is very important in the assessment of food quality and indeed human health. We are seeing many instances of where these two areas of research are combined in the field of volatilomics. In the future we are likely to see more advanced analytical techniques, machine olfaction and artificial intelligence further impact on this field to better understand and monitor food quality. Also better understanding the role of food and associated flavor compounds as a preventative medicine of the future is an important current area of research.

## Author contributions

Conceptualization: BC and ND. Writing—original draft preparation: BC. Writing—review and editing: BC, MW, and ND. All authors contributed to the article and approved the submitted version.
